# Improving facility-based care: eliciting tacit knowledge to advance intervention design

**DOI:** 10.1136/bmjgh-2022-009410

**Published:** 2022-08-19

**Authors:** Mike English, Jacinta Nzinga, Jacquie Oliwa, Michuki Maina, Dorothy Oluoch, Edwine Barasa, Grace Irimu, Naomi Muinga, Charles Vincent, Jacob McKnight

**Affiliations:** 1 Health Services Unit, KEMRI - Wellcome Trust Research Programme, Nairobi, Kenya; 2 Health Systems Collaborative, Nuffield Department of Medicine, Oxford, UK; 3 Health Economics Research Unit, KEMRI - Wellcome Trust Research Programme, Nairobi, Kenya; 4 Department of Paediatrics and Child Health, University of Nairobi College of Health Sciences, Nairobi, Kenya; 5 Center for Tropical Medicine and Global Health, University of Oxford Centre for Tropical Medicine, Oxford, UK; 6 Department of Experimental Psychology, University of Oxford, Oxford, UK

**Keywords:** Health services research, Study design, Health systems evaluation

## Abstract

Attention has turned to improving the quality and safety of healthcare within health facilities to reduce avoidable mortality and morbidity. Interventions should be tested in health system environments that can support their adoption if successful. To be successful, interventions often require changes in multiple behaviours making their consequences unpredictable. Here, we focus on this challenge of change at the mesolevel or microlevel. Drawing on multiple insights from theory and our own empirical work, we highlight the importance of engaging managers, senior and frontline staff and potentially patients to explore foundational questions examining three core resource areas. These span the physical or material resources available, workforce capacity and capability and team and organisational relationships. Deficits in all these resource areas may need to be addressed to achieve success. We also argue that as inertia is built into the complex social and human systems characterising healthcare facilities that thought on how to mobilise five motive forces is needed to help achieve change. These span goal alignment and ownership, leadership for change, empowering key actors, promoting responsive planning and procurement and learning for transformation. Our aim is to bridge the theory—practice gap and offer an entry point for practical discussions to elicit the critical tacit and contextual knowledge needed to design interventions. We hope that this may improve the chances that interventions are successful and so contribute to better facility-based care and outcomes while contributing to the development of learning health systems.

**Summary box**

- Interventions involving facility-based care may have apparently disappointing results with increasing recognition that making the changes we desire can be very difficult in complex systems.
- Theories and frameworks drawn from multiple disciplines offer means to examine complexity, but there seem few intuitive entry points that might help engage practitioners and uncover their tacit and contextual knowledge of local health systems.

- We offer ideas that may help initiate discussions with local practitioners to uncover their tacit and contextual knowledge.
- We suggest considering three core resource areas and what we term five motive forces—strategies that might be deployed to overcome system inertia to produce change. Exploring the three core resources and how to take account of the five motive forces provides a means to examine the hardware and software of local health systems as interventions are being designed.
- Such thinking may result in: broader thinking about what it will take to intervene successfully, using more creative strategies, or prompt use of more formal codesign approaches.

- Interventionists targeting the complex environment of health facilities should pay careful attention to local practitioners tacit and contextual knowledge before they start. Effective interventions will likely need to adapt over time in response to continuous learning despite the fact that adaptive intervention approaches create challenges for researchers and funders.

## Background

The clear, targeted solutions of the Millennium Development Goals era yielded major health gains in areas such as child mortality through the successful expansion of specific intervention programmes such as immunisation.^1^ These programmes achieved success in part through careful central planning, standardisation of processes and monitoring. Now reducing mortality and morbidity with highly specific interventions is becoming harder to achieve. Therefore, attention has also turned to the challenge of improving the quality and safety of care within health facilities in low-income and middle-income countries (LMICs).

Improving systems of care in facilities is proving difficult to achieve. The process of change is less predictable and a larger range of factors need to be considered in advance of implementation. Here, we focus on practitioners charged with or seeking to change facility-based care, such as programme leaders, health service managers and local leaders. We aim to provide them with an entry point to anticipating and addressing the challenges they might face. We suggest first that they should engage facility managers, senior and front-line staff, and potentially patients and communities to explore the resources available and the realities of the context they seek to change. We then offer examples of questions that might be asked to illustrate the value of eliciting local knowledge, efforts that could progress to fuller forms of codesign. Third, we illustrate in summarised form the importance of explicitly addressing the relational or ‘software’ aspects of contexts in planning for change. Throughout, we draw on theory and empirical work to describe the main challenges of making improvements in complex systems, but acknowledge that our aim is to provide an intuitive entry point to this broad and growing field.

## Interventions in complex systems

The Lancet Global Health’s 2018 report on High Quality Health Systems made it clear that any interventions are delivered within, and are part of, complex systems.^2^ Here, we take a broad view of interventions, considering them to encompass efforts to change how or by whom, care is provided and the introduction of new therapies, tools or technologies and much more. Acknowledging that we must deal with complexity when intervening means moving beyond strategies driven only by logical frameworks and overcoming specific ‘bottlenecks’.^3^

There are many resources now available to help the academic community deal with issues of complexity and implementation of interventions in health facilities. These include guidance on the research process and frameworks and theories drawn from the fields of psychology, social and organisational sciences, and human factors among others.^3–7^ They characterise, in different ways and at different levels of abstraction, some of the principal properties of human systems and can guide thinking on how to design, examine or interpret the effects of interventions.^8–13^ We believe, however, that there are few entry points to this field that help engage those in practice.

In particular we propose, as have others, that the tacit and contextual knowledge of those in practice is invaluable to effective intervention design and execution and we hope to offer a set of ideas on how to start the conversations that elicit this knowledge.^14^ More generally, we wish to promote shared thinking as a deliberate process advanced through reflection on change efforts, a form of thinking by managers that Mintzberg recognised as key to system learning, something also highlighted recently by others.^15^ Our aim is to move practitioners’ thinking beyond the idea that interventions address a single or small number of perceived system deficits that if addressed will have predictable and highly beneficial effects. For example, scaling up access to faster and more advanced diagnostics for tuberculosis offers a putative solution to a long-standing problem. However, work to examine the context in which these technologies were being introduced identified a multiplicity of limiting factors including inadequate health worker skills, poor integration of sample collection into workflows and a long-standing and pervasive clinical norm that tuberculosis in children needs only a clinical diagnosis.^16^ All these and others need to be tackled using different strategies to promote use of this diagnostic. Our hope is that incorporating a better understanding of contexts will help reduce the risk of intervention failure.^17^

To draw out this contextual and tacit knowledge, we need to ask the right questions. Below, we outline potentially useful starting points. These might guide initial discussions during meetings or workshops with local healthcare managers, front-line staff, patients and communities who may be affected by the intervention. Inviting such people to critique the intervention design early in its development and identifying key challenges to its effectiveness may substantially influence the initial design or cause us to rethink it completely. This can provide an opportunity to reflect on whether the initially proposed intervention is actually the priority and a basis on which go or no-go decisions can be made. Ideally, addressing these initial questions should be the start of a shared learning journey, but our more limited aim here is to propose its starting point.^9 14^

## Assessing capacity for change: three core resources

**Figure 1:**
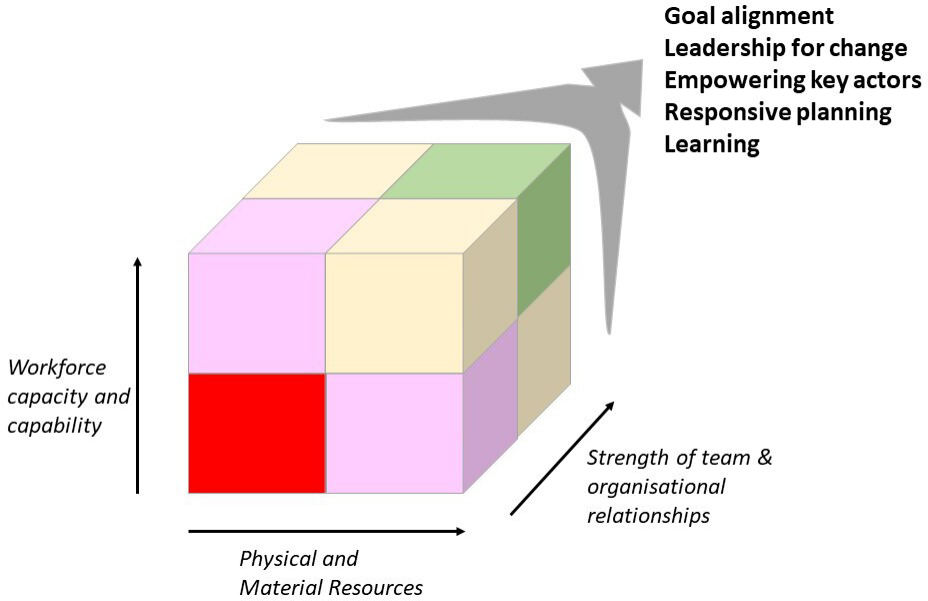
Facility level improvement—the quality and safety cube. In many LMIC the quality, safety and thus outcomes of facility-based care are undermined by challenges in three core resource areas, inadequate physical or material resources, deficits in workforce capacity and capability, and poor team and organisational relationships. As a result, local facilities operate within the red zone of the cube. Effective interventions that address one or two resource issues may result in unidimensional or bidimensional improvements. However, in weak systems, all three areas need strengthening. Addressing resource inadequacies typically needs to be accompanied by efforts to create and align motive forces if the system status quo is to be changed. Learning across the system and recognising progress can help address unanticipated challenges or consequences and build momentum. (LMIC, low-income and middle-income country).

We draw on experience of using multiple approaches to understand facility-based care and to design and examine the response to interventions for this report. For example, our work has examined implementation experiences,^18^ the work and influence of clinical and facility leaders,^19 20^ how theory might inform intervention design,^21^ how norms shape practice,^22^ and efforts to diagnose micro, meso and macro health system challenges.^23 24^ This work leads us to suggest first considering three foundational questions on the resources available in the contexts targeted for change, and how these might impact the delivery and outcomes of an intervention. In many LMICs, the quality, safety and thus outcomes of facility-based care are undermined by the challenges in three core resource areas: inadequate physical or material resources, deficits in workforce capacity and capability, and poor team and organisational relationships. These resource areas can be thought of as forming three dimensions. In figure 1, we illustrate how some facilities may be thought of as operating within a red zone with major weaknesses in each dimension. We acknowledge that these three dimensions, and their division into simple binary categories, are a major simplification of what are more nuanced gradations and many other attributes. However, the aim of this illustration is to promote more holistic thinking among practitioners. For example, facilities in this red zone will probably need strengthening in all three areas to support a successful intervention or deliver high quality safe care as we illustrate in panel 1 (represented in figure 1 as a move to the green zone). Interventions in these settings that address only one or two resource dimensions may result in only unidimensional or bidimensional improvements (represented by a shift in figure 1 towards the pink or yellow zones of the cube, respectively). In other systems, the starting position may be stronger on one or more dimensions. For example, physical and material resource limitations are much less likely to be a concern in facilities in high income countries. Achieving success (reaching the green zone) in such settings may be achieved by judicious diagnosis of the problems and more specific strengthening in one or two dimensions. We provide some more specific illustrations of these three resource dimensions below before considering how achieving change typically needs to go beyond ‘core resources’ to address motive forces.

Before beginning the phase of inquiry focused on the facility setting itself it is important to consider whether the wider health system context and institutional arrangements can support the change being planned or could do if good evidence of effect is produced. Identifying macro level challenges that must also be overcome is just as important, and may also inform prioritisation or go/no-go decisions, but is beyond the scope of this report. To examine the three, core resource dimensions, and focusing on intervention at the mesolevel and microlevel, we propose starting with broad questions as an entry point for open and more detailed discussion:

### Are or will there be sufficient infrastructure and consumable material resources?

Many low-resource facilities struggle with inadequate space, layouts not suited to care processes and inadequate basic infrastructure such as power, water and sanitation. Supplies of basic consumables, drugs and, as recently highlighted, personal protective equipment (PPE) may be intermittent or inadequate for needs. An intervention may address one problem such as improving oxygen supplies and skilled high-dependency care but fail to improve outcomes if needed antibiotics are missing, power is intermittent and soap and water for hand washing are not always available.

### Is there, or will there be, sufficient capacity and capability in the workforce?

Interventions often seek to improve care by addressing limited health worker capability (the ability or skill to do something). Examples include training and increasingly use of technological aides such as decision support or telemedicine to enhance skills and practices. Such interventions may also be accompanied by temporary supply of new diagnostic tools or resources. Despite often very obvious inadequacies in staff numbers, for example nurse to patient ratios of 1–15 or more,^25^ interventions almost never seem to address staff numbers (capacity) as a critical determinant of effectiveness. Nor do those planning interventions pay sufficient attention to whether the change they propose will increase the work that needs to be done. Even when this appears marginal such as improving documentation or including a new bedside test such as pulse oximetry, these often require small additional steps in clinical practice and additional management work spanning: centralised procurement, checking and handover of ward stocks, and ensuring equipment functionality and maintenance.^26^

### Are all the people, groups and teams directly and indirectly affected by the intervention likely to support and coordinate their efforts to effect any anticipated changes in patterns of work or responsibilities?

Most facility delivered care results from the work of multiple individuals in diverse roles. Relations between all these people determine the nature of this care, and how different team members organise themselves around a new activity will impact the success of any intervention (eg, issues explored in detail in normalisation process theory).^27^ In most facilities therefore, local interpersonal and interprofessional relations, and team and facility management and leadership are key to an intervention’s success. In our own work, this is apparent from examples exploring the adoption of bedside technologies, laboratory-based diagnostics and broader efforts to promote the use of multiple, evidence-based guidelines (see panel 1).^16 19 26^ Although such relationships are often acknowledged as critically important post hoc in reports on intervention effectiveness they seem surprisingly rarely addressed as a deliberate part of interventions.

In panel 1, we illustrate how these three core resource areas may interact and how the effectiveness on health outcomes of discrete interventions targeting severe acute malnutrition (SAM) would be limited. Having highlighted core resource dimensions we turn attention now to the efforts that may be required to initiate and embed change in facilities to promote intervention effectiveness.

**Panel 1: illustration of how challenges within the three core resource dimensions and their interaction affect the success of programmatic interventions, the example of care for SAM**

The WHO and UNICEF have promoted use of the 10 steps for care of children with SAM for over 30 years. Hospitals might be expected as a default to be able to meet the key physical and material resource requirements for managing a common, serious condition. These span accommodation that can keep affected children warm at night and that supports infection prevention together with essential material resources enabling temperature measurement, testing for hypoglycaemia and anaemia and specific milk, feed and micronutrient therapies. Indeed, UNICEF works in many countries, and for many years has supplied facilities with prepackaged milk formula and solid feed preparations. A logical and widely used intervention to complement these material resources is dissemination of guidelines ideally as part of pre-service and in-service training to build capability among health workers to diagnose and manage SAM to improve outcomes. This approach to intervention, further enhanced by monitoring and feedback of quality of SAM care processes in hospitals, has been used in Kenya in various forms for over 15 years. However, while scaling up of guidelines and training has occurred with improvements in some practices, outcomes have remained largely unchanged.^52 53^

Factors contributing to persistently poor outcomes of care for SAM span the three core resource areas. Thus, after 15 years even larger hospitals still face major challenges in providing a safe physical environment with persistent water, sanitation and infection control challenges compounded by undersupply of key materials, for example those enabling bedside blood glucose testing.^54 55^ There is some improvement in skills (capability) of those who initiate clinical management. For example, in appropriate prescribing of specific milks or feeds, but the system relies on this being done by the most junior front-line staff. These personnel begin with limited capability and rarely gain expertise because of rapid rotation through different departments, while they have very limited supervision as there are too few senior staff.^56 57^ At the same time, too few nursing staff struggle to monitor sick children and consistently deliver even the most essential therapies.^25^ Both reflect problems of inadequate workforce capacity that have changed little over many years. A consequence of low staffing numbers is limited interaction between medical and nursing personnel and unclear roles for nutritionists, where these are actually available. Poor collaboration and teamwork contribute to delays in executing and sustaining treatment plans, and failings in recognising and dealing with complications. At the same time, mothers of children with SAM are often poorly engaged as carers, reflecting poor communication with staff, and may even be stigmatised as bad parents.^58^

Improving outcomes of SAM requires that these multiple challenges that span all three core resource dimensions are tackled holistically. This needs sustained effort with potentially even more far-reaching strategies required to improve outcomes after hospital discharge.^59^

## Drivers of change: the five motive forces

Improvement typically requires changes in behaviour by many different health workers spanning leaders, managers and front-line workers and often by patients. Inertia is built into these complex social and human systems with ways of behaving produced and sustained by a complex web of deeply embedded and interacting social influences.^28^ Overcoming the inertia in these systems requires new forces. This may ultimately mean trying to change elements of the organisational, institutional or policy environment, areas that may seem well beyond the apparent boundaries of a specific health facility intervention. When considering change we must be aware of the formal and informal rules that guide behaviours. This can mean thinking about power in its many forms and which individuals or groups might exercise their influence over an intervention,^29–31^ and how an intervention might require practices that have become deeply established over time might need to change.^32^ To emphasise the idea of overcoming inertia and fostering change, we use the term motive forces rather than a term such as lever which conjures predictable, mechanical images of action. We feel it is important to remain alert to the hidden forces that shape events and that may result in unexpected consequences.

The five motive forces we consider aggregate many elements of existing frameworks, for example, from behavioural science,^5^ implementation science^7^ and health systems thinking.^33^ We acknowledge our approach is derived to serve practical ends from a large and diverse body of important work spanning goal theory,^34^ identity theory,^29^ health policy and organisational research,^35 36^ work on leadership in health systems^37 38^ and motivation.^39^ Our aim is not to propose a substitute for these ideas but use them to help practitioners to reflect on how their intervention affects and is affected by others and therefore how local understanding may help them make adaptations that may promote success. The five motive forces linked to broad questions are:

1. Goal alignment and ownership: Arguably, ensuring all key stakeholders are aligned with the goal of an intervention is a fundamental step influencing everything from organisational responses to individuals’ motivation. How will the approach proposed ensure the goals of the intervention are considered important by everyone involved in, or affected by, the changes being made? How does the intervention ensure that they commit individually, as teams and as organisations, to achieving these goals?
2. Leadership for change: Which individuals or teams are expected to lead the change on a day-to-day basis? Are they appropriately positioned in the system/facility to do this (eg, do they have any necessary formal authority or informal power)? How will these people, typically at the mid-level of facility organisations (eg, senior health workers or department heads), gain any needed skills in strategic use of communication, coaching and problem-solving skills so they can be both effective and create the trusting relationships needed to foster change?
3. Empowering key senior actors: The mid-level change leaders we explore in the question above will need the support of more senior managers who run whole health facilities and potentially those at even higher levels of the health system. Are these more senior personnel identified, engaged and themselves empowered and willing to support the change through new policy or by tackling bureaucratic hurdles so they in turn empower those at local level? How will these key actors be recognised for achievements when changes occur so the momentum for change is sustained over time at multiple levels of the health system?
4. Responsive planning and procurement: How will any infrastructural, ongoing material or community needs (identified while considering the three core resources) be met? Are mechanisms in place to leverage new resources that go beyond any short-term external project support to meet longer-term or unanticipated and emergent needs? Will resourcing a new initiative divert existing resources from elsewhere causing a new set of problems?
5. Learning for transformation: How will evaluation and learning take place locally to identify and celebrate successes, identify challenges (including unintended consequences) and strengthen local team-working through ongoing co-production of solutions? How might local learning lead to wider shared learning that can harness the power and enhance the capability of peers and networks?

Considering each of these motive forces prompts questions on how to create the conditions that promote the desired change and who may need to be co-opted into the process of change. This thinking should complement that on the three core resource areas outlined above (table 1 and see an example in panel 2). Interventions that address a context’s actual resource needs and effectively deploy motive forces may, we suggest, help make interventions more effective within complex systems (figure 1).

**Table 1:** Inspired by practical tools, such as the Business Canvas and Lean Canvas, we provide examples of generic questions or areas of enquiry that may help begin to uncover the status of the three core resources and five motive forces in facilities forming the context of a proposed intervention^60^

**Physical and material resources** Is the basic infrastructure sufficient to support the intervention/change? If not are any changes needed to physical layouts? If so who might these affect? Are all the basic tools and materials consistently) available to offer care as a platform for any intervention/change? If there are infrastructure, layout or material deficiencies what needs to be done/added and who will do this, how and who will bear the cost? Will the intervention/change require new resources? How will these be provided? Will these incur additional costs? How will they be sustained beyond any project? For technologies how will these be maintained or disposed of?		**Workforce capacity and capability** Are there sufficient staff to provide expected forms of routine care now? if not then which personnel/skills are inadequate? How will an intervention/change be impacted by any existing personnel shortages and can these shortages be addressed? Does the intervention/change comprise ‘new work’? who will do this work? What other forms of work might not be done if new work is prioritised? Does it require new skills? Who needs to have these skills and how will they be provided? How will new skills be retained or spread during and after any project? How will desired skills in practice be monitored? What ‘shortcuts’ or ‘workarounds’ can be anticipated as workers assimilate the intervention/change?	**Relationships** Who is involved in delivering the intervention/change and which positions/people would have any supervisory or management responsibility? How are these people in the facility organised in formal roles/teams? Are there important informal groupings? How well in general do these people/groups work together currently? Are there specific professional groups or parts of the facility that may need to collaborate to deliver the intervention/change, what are relationships like between these different parties? How might the intervention/change affect existing formal and informal organisational structures, routines or roles? How might this be perceived by different groups? Have there been previous intervention/change initiatives? What affected their success?	
**Goals** Are the specific intervention/change goals clear and important to people from top to bottom of the system? Are there clear, possibly more important competing priorities? What work needs to be done to gain agreement on goals?	**Action team** Who in formal or informal leadership positions will need to drive the intervention/change? How might they be supported to navigate existing hierarchies? Why might joining this leadership team be attractive? How will the interpersonal and professional skills of this team be developed?	**Organisational support** What processes might be used to engage senior and mid-level managers to plan for the intervention change? What room do they have to adapt a standard plan? How, will authority be delegated and support given in practice to front-line teams? Is support of other influential stakeholders such as professional regulators needed? How will success be recognised or other incentives used?	**Responsiveness** How will senior managers be kept informed of intervention/change success and impacts? Do senior managers have the capacity to mobilise and deploy modest resources to address local resource or personnel needs as they arise? Are there longer term needs for procurement and planning? What advice and support may be needed to support this? How will success be recognised or other incentives used?	**Learning** What monitoring and evaluation is proposed, is it feasible in the long term, and how does it engage and inform decision-making? How might information be shared across settings to help national managers identify challenges, make and test course corrections? Who is taking on the work of such learning, do they have the time and the skills to do this and who is responsible for the process?

A large, blank print version of this type of representation might be used to generate ideas in a workshop, or thematic areas might be tackled as individual topics. Aggregating key findings in such a matrix may help identify cross-linkages or dependencies that inform intervention thinking.

**Panel 2: example of findings (see accompanying table) that might arise in an initial discussion of a hypothetical programme and exploration of the three core resources and the five motive forces- The National Outreach to Advance Health and Accelerate Respiratory Care programme**

This aims to improve respiratory care in 12 general hospitals in a low-resource setting that have not previously had any form of high-dependency or critical care. Intervention inputs were planned to include as follows: (1) installing a centralised oxygen supply with funding to develop a four-bed high-dependency unit (HDU) with Continuous Positive Airway Pressure (CPAP) machines, patient monitors and sufficient oxygen consumables to last an initial 24 months; (2) specific training for one senior physician and six nurses on the oxygen system, CPAP devices and on more general provision of HDU care with the expectation that the nurses will simultaneously act as respiratory technicians/therapists; (3) small-scale funding to enable this local team to cascade training across the hospital in the initial 12 months and (4) funding to support a specialist from a tertiary care setting to conduct quality assessments linked to mentorship visits on a quarterly basis in the initial 12 months.

**Table:** 

Discussions with local managers, departmental leaders and front-line staff yielded the following immediate questions about The National Outreach to Advance Health and Accelerate Respiratory Care (NOAH's ARC) programme.				
**Physical and material resources.** What level of support will actually be provided for oxygen and monitoring related consumables? Will use of these need to be ‘rationed’ and only permitted on the high-dependency unit (HDU)? What new costs will the hospital incur to sustain procurement of an expanded set of oxygen consumables used at higher volumes? Will a larger generator be needed for periods when mains electricity is interrupted to power machines? If an area of the surgical ward is refurbished and repurposed as a 4-bed HDU, what impact will this have on surgical care? Will other wards also have new areas designated for those needing oxygen and better monitoring or should all those needing oxygen go to the HDU?		**Workforce capacity and capability.** Capability (training) seems to be addressed but hospitals will get no new nurses, Tto ensure one of the six trained nurses is on the HDU 24/7 will they have to work 12-hour shifts and increase working hours? Will they tolerate this? What happens in case of absence? Policy on HDU indicates 1 nurse to 3 patients, previously when offering only low-flow oxygen policy allowed for 1 nurse to 6 patients. Is it reasonable to have only one nurse working alone for 4 HDU beds? general wards will lose nurses, increasing their workloads adversely affecting staff and patients. If only one physician is fully trained is it safe to operate the HDU if this physician is unavailable? Or will new physicians need to be recruited and paid for?	**Organisational relationships.** When the trained physician is not available will the ‘specialist nurses’ trust the medical advice of less well-trained physicians? Might this result in junior clinicians avoiding the HDU as they become fearful of making mistakes? Could this lead to emergence of a ‘them and us’ situation especially at vulnerable times such as nights and weekends? Will relationships between the HDU nurses and wider hospital nursing body sour if they feel those on HDU are getting special treatment such as paid overtime? There have been great difficulties in negotiating referrals with the referral level hospital with local staff feeling their efforts are not respected and even feeling patronised, how will productive relationships be developed beyond respiratory care?	
**Goal alignment.** Improving oxygen supplies is a local priority but not confined to respiratory illnesses, post-operative, maternity and neonatal care are also priorities. Support for equipment, resources, staff training and maintenance should extend to all these areas.	**Action team** There is one local physician, s/he and a senior nurse could be key focal points. They will have to work with senior staff to reallocate nurses and liaise with the biomedical engineering department. They will need to negotiate with surgical team leaders to redesign the allocated space, will they become a separate organisational unit (eg, for resource and personnel management) or be part of the surgical or medical unit? The physician already runs the medical ward.	**Organisational support** How will the new HDU team leaders engage with senior facility managers, will they need to join hospital management committees? How many? Who will order and manage the equipment and consumables provided, will they be given training to do this and who will be involved in long-term resource planning to support the HDU? Under what circumstances can the senior team allocate resources to other priority areas? Who will develop local guidelines for admission to the HDU and manage disagreements?	**Responsiveness** As provision of HDU care is not currently part of national/regional policy and planning how will this expansion of service delivery be allocated budgets to enable sustained HDU support? Current health information systems are not designed to capture HDU workloads and outcomes, how will this be addressed to support planning and management? What possibilities are there for employing additional staff to support the HDU?	**Learning** What form will support from the regional referral centre take? Will it only involve the expert visiting the facility? Or will HDU staff visit the tertiary centre or other units to share practice ideas? Is advice available 24/7 in emergencies, through what mechanism? Is someone responsible for capturing learning on implementing HDU care and sharing this with policy makers and planners?

## Putting it into practice

Even those interventions that may appear the simplest, such as the introduction of a single new diagnostic test or device represent a change in processes of care. To yield a health benefit the change process must be successful, often needing to work together with other factors that help co-produce this benefit (see panels 1 and 2). For those wishing to explore the three core resource areas to uncover local contextual and tacit knowledge we suggest techniques more commonly associated with quality improvement or patient safety might be used. For example, the co-creation of a process map or an examination of the work system to characterise existing practices and examine how intervention may need to change things may be useful.^16^ This might be done in a workshop or series of meetings or discussions involving managers, front-line workers, those offering supportive services spanning laboratory personnel to cleaners and potentially patients. Facilitators can draw on much available guidance on the conduct of such exercises.^40 41^ We offer examples of questions that might be asked alongside or as part of such events in table 1 and, in panel 2, we offer a hypothetical illustration of what such exploration might reveal for a service redesign intervention. (A blank ‘canvas’ might be used to structure exploration of other interventions or contexts). It is important to recognise that very important information may be gained from informal conversations and during other exercises such as facility ‘walk throughs’ where the intervention is imagined with local teams.^16^ This may be especially useful for exploring the relationships between groups, teams and professions.^16^ More formal codesign approaches can also be used if time and resources are available^42 43^ with reflective exercises helpful when considering the five motive forces that might support change.^44^

## Conclusion

Much has been written on the need for better implementation and the importance of context.^45–48^ Despite this, it still seems rare for interventions targeting facilities within weak health systems to deliberately identify and tackle challenges in all three core resource areas simultaneously. Many interventions deal with only one or two issues or resource areas. For example, interventions focus on a single area of health workers’ technical skills, only provide a new tool or technology or only offer senior staff management training. While absence of very specific resources (eg, particular technologies) can be a problem this is very rarely the only problem.^26^ The predominant focus on workforce capability in particular, for example, by enhancing individuals’ skills through training or provision of decision support, is very rarely accompanied by efforts to tackle the major and persistent problems of serious workforce shortages (capacity). Although poor management and leadership are often retrospectively identified as causes of intervention failure it is surprisingly uncommon for interventions to include deliberate efforts to improve organisational relationships or other elements of ‘intangible software’.^49^ As we emphasise, it is also critical for intervention designs to go beyond even these three core resources.

Our practical perspective proposes as a starting point that five motive forces are also considered as part of careful, open discussions on a potential intervention. This may mean those proposing and often invested in an intervention need to be prepared to see its design and intent revised to promote effectiveness and potential sustainability, or even postponed until critical underlying challenges can be overcome. The research community has learnt that without carefully identifying existing challenges even seemingly well considered, carefully conducted and sometimes generously supported interventions targeting facility-based care can fail to achieve their expected impacts.^17 41 50^ The importance of working with practitioners to prioritise needs and design interventions that recognise local causal pathways has recently been highlighted.^9 14^ What we hope to contribute are practical questions to guide the earliest stages of this process. These may suggest the need for more specific, formal codesign strategies.^40 41 51^ Unfortunately, funding models for such codesigned research approaches are uncommon. In fact, priorities for intervention are often dictated by specific funder interests (eg, in technologies) or development objectives (eg, expanding access to a specific form of care). Interventions directed at facilities continue therefore, to comprise a rather narrow set of strategies and it may be difficult to get support to employ less familiar, more dynamic and adaptive intervention approaches. We therefore join others in calling for intervention and learning to be parallel activities. We hope the entry point of considering the three broad resource areas and broader motive forces, spanning both the hardware and software of systems, may move people beyond relatively well-worn implementation paths. Such approaches may not lend themselves to neatly designed experiments but may help foster the development of broader learning health systems and ultimately prove more effective in improving facility-based care and outcomes.^3 44^

## Data Availability

No data are available.
